# Estradiol and Testosterone Concentrations Among Thai Transgender Women in a Transgender-Led, Integrated Gender-Affirming Care and Sexual Health Clinic: A Real-World Analysis

**DOI:** 10.1089/trgh.2021.0049

**Published:** 2022-11-29

**Authors:** Akarin Hiransuthikul, Rena Janamnuaysook, Pintusorn Getwongsa, Jitsupa Peelay, Kritima Samitpol, Tidarat Amatsombat, Phongdanai Chumnanwet, Artsanee Chancham, Jiratchaya Kongkapan, Jeeranuch Rueannak, Linrada Himma, Peevara Srimanus, Nipat Teeratakulpisarn, Matthew Avery, Tanyaporn Wansom, Stephen Mills, Reshmie A. Ramautarsing, Nittaya Phanuphak

**Affiliations:** ^1^ Institute of HIV Research and Innovation (IHRI), Bangkok, Thailand.; ^2^ Department of Preventive and Social Medicine, Faculty of Medicine, Chulalongkorn University, Bangkok, Thailand.; ^3^ Center of Excellence in Transgender Health (CETH), Chulalongkorn University, Bangkok, Thailand.; ^4^ FHI 360 and LINKAGES, Bangkok, Thailand.

**Keywords:** estradiol, feminizing hormone, gender-affirming care, testosterone, transgender women

## Abstract

**Purpose:**

Feminizing hormone therapy (FHT) is used by many transgender women as a pharmacological method to mitigate gender dysphoria. However, information on hormone concentrations among those who use FHT is lacking. We aimed to determine the proportion of Thai transgender women who were using FHT who had hormone concentrations within target ranges in a real-world clinic setting.

**Methods:**

Transgender women who attended Tangerine Clinic in Bangkok, Thailand, reported current use of FHT at clinic entry, and tested for both blood estradiol (E2) and total testosterone (TT) concentrations were included in the analysis. Hormone target concentrations were defined as 100–200 pg/mL for E2 and <50 ng/dL for TT.

**Results:**

Of 1534 transgender women included, 2.5% had undergone orchiectomy, and 524 (34.2%) had any hormones within target concentrations. Median (interquartile range) E2 and TT concentrations at baseline were 29 (14.3–45.3) pg/mL and 298.5 (22–646) ng/dL, respectively. Among those who had any hormones within target concentrations, 28 (1.8%), 11 (0.7%), and 485 (31.6%) had both hormones, only E2, and only TT within target concentrations, respectively. Among 1010 (65.8%) transgender women who had neither hormone within target concentrations, 989 (64.5%) and 21 (1.4%) had suboptimal and supraphysiological E2 concentrations, respectively. Among those who came to at least one follow-up visit (*n*=302), 165 (54.6%) transgender women managed to achieve or maintain either hormone within target concentrations.

**Conclusion:**

One-third of Thai transgender women who were using FHT had any hormones within target concentrations at baseline in this real-world setting study. Most transgender women who had neither hormone within target concentrations had suboptimal rather than supraphysiological E2 concentrations. More than half managed to achieve or maintain at least one hormone concentration within target concentrations at follow-up visits, suggesting a positive effect from attending a trans-led, integrated gender-affirming care and sexual health service.

## Introduction

Gender dysphoria refers to discomfort or distress that is caused by a discrepancy between a person's gender identity and sex assigned at birth.^1^ Feminizing hormone therapy (FHT) is among the options, and the recommended pharmacological method, to mitigate such discrepancy among transgender women. The goal of FHT is to induce secondary sex characteristics of the female gender and reduce sex characteristics of the male gender.^2^ Although there are several regimens of FHT, they can be broadly categorized into three groups: estrogen (e.g., estradiol valerate and estradiol hemihydrate [EH]), antiandrogens (e.g., cyproterone acetate, spironolactone, GnRH agonists—goserelin, buserelin, and triptorelin—and 5-alpha reductase inhibitors—finasteride and dutasteride), and progestins.^1^ Differences in accessibility to hormone regimens between regions, diversities in individual's goal, and lack of clinical trials data have contributed to various hormone regimens.^1^

In Thailand, both over- and under-the-counter hormones for off-label purposes are easily accessible.^3^ As many Thai transgender women may seek social acceptance through physical beauty, it is unsurprising that most Thai transgender women have been using FHT for an average of 10 years.^4,5^ Unfortunately, transgender women usually take FHT based on their transgender peers' experiences without proper monitoring.

FHT can increase the risk of diseases or conditions such as venous thrombosis, cholelithiasis, transaminitis, and hypertriglyceridemia.^1^ Therefore, it is recommended that transgender women who used FHT should be routinely monitored.^1,2^ The target hormone concentrations for transgender women who are using FHT are testosterone concentrations below the upper limit of normal cisgender women (CGW) range (<50 ng/dL) and estradiol (E2) concentrations within a premenopausal CGW range (100–200 pg/mL), with an emphasis on avoiding supraphysiological concentrations, which may lead to increased risk of adverse events.^6^

To add to limited data around FHT efficacy among Asian transgender women, we aimed to determine the proportion of Thai transgender women who were using FHT who had hormone concentrations within target ranges in a real-world clinic setting.

## Methods

### Enrollment of participants

Tangerine Clinic in Bangkok, Thailand, is among the first transgender-led sexual health clinics in Asia. Tangerine Clinic offers integrated gender-affirming care and sexual health care, including hormone counseling, administration, and monitoring. Managed by trained transgender staff and gender-sensitive medical professionals, Tangerine Clinic has already served up to 4000 transgender women since its opening in November 2015. Tangerine Clinic's protocol for transgender care was developed based on the World Professional Association for Transgender Health Standard of Care for the Health of Transexual, Transgender and Gender Nonconforming People version 7, the Center of Excellence for Transgender Health Guidelines for the Primary and Gender-Affirming Care for Transgender and Gender Nonbinary People, and the Asia Pacific Transgender Network Blueprint for the Provision of Comprehensive Care for Trans People and Trans Communities in Asia and the Pacific, with particular modifications explicitly made for Asian transgender individuals.^2,7–9^ Transgender women who attended Tangerine Clinic, reported current use of FHT at clinic entry, and tested for both blood E2 and total testosterone (TT) concentrations between November 2015 and June 2020 were included in the analysis. Although several terms can be used to identify transgender spectrum, the term “transgender women” is used throughout this article for readability purposes.

### Ethical approval

The study protocol was approved by the Institutional Review Board (IRB) of the Faculty of Medicine, Chulalongkorn University, Bangkok, Thailand (IRB No. 158/56), for the collection, storage, and analysis of data at Tangerine Clinic. According to the approved protocol, consent was not required from the client for anonymous data collection.

### Hormone concentrations and metabolic parameters

E2 and TT concentrations were measured by the Elecsys Estradiol III and Elecsys Testosterone II electrochemiluminescence immunoassay (Roche Diagnostics GmbH, Mannhein, Germany), with reference intervals as follows: E2, 25.8–60.7 pg/mL for cisgender men (CGM), 12.4–233 pg/mL (follicular phase), 41–398 pg/mL (ovulation phase), and 22.3–341 pg/mL (luteal phase) for CGW; TT, 290–800 ng/dL for CGM and 6–80 ng/dL for CGW. Based on Hembree et al.,^6^ Tangerine Clinic's protocol set the target E2 concentration at 100–200 pg/mL and target TT concentration at <50 ng/dL. All transgender women who did not achieve target concentrations were advised to adjust their hormone regimens accordingly. Multidisciplinary staff, including doctors, nurses, and transgender women peers, were involved in providing care and counseling to individual transgender women clients. Regular follow-up visits of every 3–6 months in the 1st year were scheduled to check any potential adverse events, adherence issues, other health-related concerns, perform a physical examination, and monitor hormone concentrations. Baseline and available 12-month period of follow-up E2 and TT concentrations during the analysis period were assessed. Self-administered written questionnaires were used to assess demographic data and risk behaviors. Available baseline metabolic parameters (hematocrit, hemoglobin, mean corpuscular volume, white blood cells, plate counts, creatinine, alanine aminotransferase, and lipid profiles) were assessed.

### Statistical analysis

Statistical analysis was performed using Stata 13 (StataCorp LP, College Station, TX). Baseline characteristics, hormone concentrations, and other metabolic parameters were summarized as median (interquartile range [IQR]) for continuous variables and frequency (percentage) for categorical variables; and were categorized by those who had any hormones within target concentrations and had neither hormone within target concentrations at baseline. Mann–Whitney *U* and chi-square or Fisher's exact test were used to test significance between groups as appropriate. Regression methods were used to access the linear trend changes over follow-up visits. Multivariable logistic regression was built from baseline covariates associated with having both hormones within target concentrations in univariable regression. Statistical significance was defined as *p*-value of <0.05.

## Results

### Participant characteristics

Among 3480 transgender women who attended Tangerine Clinic between November 2015 and June 2020, 2171 reported using FHT at their clinic entry. A total of 1534 transgender women were tested for both E2 and TT concentrations at baseline, and were included in the analysis. Median age (IQR) was 23 (20–28) years, 1020 (91.6%) identified themselves as transgender women, 19 (1.7%) as male, and 75 (6.7%) as female; 2.5% had undergone orchiectomy, and 5% were HIV positive (Table 1).

**Table 1.** Baseline Characteristics of 1534 Transgender Women Included in the Analysis

Characteristics	Overall (*n*=1534), *n* (%)	Hormone(s) within target concentrations (*n*=524), *n* (%)	Neither hormone within target concentrations (*n*=1010), *n* (%)	*p*
Median age (years)	23 (20–28)	23 (20–27)	24 (21–28)	0.01^*^
Median body weight (kg)	59 (54–66)	58 (53–65)	60 (54–67)	0.004^*^
Gender identity				0.11
Male	19/1114 (1.7)	6/397 (1.5)	13/717 (1.8)	
Female	75/1114 (6.7)	35/397 (8.8)	40/717 (5.6)	
Transgender women	1020/1114 (91.6)	356/397 (89.7)	664/717 (92.6)	
Bachelor's degree or higher	716/1498 (47.8)	230/511 (45.0)	486/987 (49.2)	0.12
Occupation				0.01^*^
Unemployed	149/1492 (10.0)	55/506 (10.9)	94/986 (9.5)	
Student	453/1492 (30.4)	177/506 (35.0)	276/986 (28.0)	
Employed, other than sex worker	723/1492 (48.5)	229/506 (45.3)	494/986 (50.1)	
Sex worker	167/1492 (11.2)	45/506 (8.9)	122/986 (12.4)	
Multiple sex partners	697/1252 (55.7)	198/416 (47.6)	499/836 (59.7)	0.01^*^
Underwent orchiectomy	38/1526 (2.5)	35/521 (6.7)	3/1005 (0.3)	<0.001^*^
HIV positive	72/1429 (5.0)	11/483 (2.3)	61/946 (6.5)	<0.001^*^
Median estradiol (pg/mL)	29 (14.3–45.3)	25.0 (5–66.8)	29.9 (19–41.8)	0.12
Median total testosterone (ng/dL)	298.5 (22–646)	13 (5.5–22.6)	530 (301–784)	<0.001^*^
Median years of hormone use^a^	3.9 (1.2–7.4)	3.2 (0.8–6.3)	4.0 (1.4–8.0)	<0.001^*^
Hormone regimens^b,c^				0.48^d^
Combined regimen	323/1105 (29.2)	117/383 (30.6)	206/722 (28.5)	
EE + CPA oral	313/1105 (28.3)	113/383 (29.5)	200/722 (27.7)	
EE + DRSP oral	9/1105 (0.8)	3/383 (0.8)	6/722 (0.8)	
EE + GSD oral	1/1105 (0.1)	1/383 (0.3)	—	
Single regimen	782/1105 (70.8)	266/383 (69.5)	516/722 (71.5)	
Estrogen	367/1105 (33.2)	129/383 (33.7)	238/722 (33.0)	
17β-estradiol gel	18/1105 (1.6)	5/383 (1.3)	13/722 (1.8)	
EC oral	5/1105 (0.5)	4/383 (1.0)	1/722 (0.1)	
EV injection	65/1105 (5.9)	21/383 (5.5)	44/722 (6.1)	
EV oral	270/1105 (24.4)	95/383 (24.8)	175/722 (24.2)	
EH oral	9/1105 (0.8)	4/383 (1.0)	5/722 (0.7)	
Antiandrogen	365/1105 (33.0)	125/383 (32.6)	240/722 (33.2)	
Bicalutamide injection	1/1105 (0.1)	—	1/722 (0.1)	
CPA oral	360/1105 (32.6)	125/383 (32.6)	235/722 (32.6)	
Finasteride oral	2/1105 (0.2)	—	2/722 (0.3)	
Spironolactone oral	2/1105 (0.2)	—	2/722 (0.3)	
Progesterone^e^	50/1105 (4.5)	12/383 (3.1)	38/722 (5.2)	
Self-reported use of ARVs^f^	42 (2.7)	12 (2.3)	30 (3.0)	0.44

Continuous variables were demonstrated as median (IQR).

^*^ Indicates *p*-value of <0.05.

^a^ Data were available among 1104 participants.

^b^ Information on hormone regimens was available among 1105 of 1534 transgender women.

^c^ Per oral route except noted.

^d^ Compared single versus combined hormone regimen.

^e^ All were using hydroxyprogesterone caproate injection.

^f^ Eight transgender women were using ART (all but one were using TDF/FTC/EFV, the remaining was using TDF + FTC + DTG) and 34 transgender women were using PrEP (daily TDF/FTC).

ART, antiretroviral therapy; ARVs, antiretroviral agents; CPA, cyproterone acetate; DRSP, drospirenone; DTG, dolutegravir; EC, estradiol conjugate; EE, ethinyl estradiol; EFV, efavirenz; EH, estradiol hemihydrate; EV, estradiol valerate; FTC, emtricitabine; GSD, gestodene; IQR, interquartile range; PrEP, pre-exposure prophylaxis; TDF, tenofovir disoproxil fumarate.

#### Hormone regimens

Fourteen different FHT regimens were recorded: 29.2% were combined hormone regimens (all were ethinyl estradiol-based regimens) and 70.8% were single hormone regimens. Estrogen-based regimens accounted for 62.4% of all FHT use. These include ethinyl estradiol + cyproterone acetate (*n*=313; dosage ranges 1 tablet occasionally to 21 tablets/day), ethinyl estradiol + drospirenone (*n*=9; 1–2 tablets/day), ethinyl estradiol + gestodene (*n*=1; 1 tablet/day), 17β-estradiol gel (*n*=18; 3 times/week to once daily), estrogen conjugate oral (*n*=5; 1–2 tablets/day), estradiol valerate injection (*n*=65; twice a week to once a month), estradiol valerate oral (*n*=270; 268 participants took 1/4–4 tablets/day, and the remaining 2 participants took 1 tablet occasionally and once weekly), EH oral (*n*=9; 1–8 tablets/day).

#### Hormone concentrations

Overall, median E2 and TT concentrations were 29 (14.3–45.3) pg/mL and 298.5 (22–646) ng/dL, respectively. A total of 524 (34.2%) TGW had any hormones within target concentrations: 28 (1.8%), 11 (0.7%), and 485 (36.6%) had both hormones, only E2, and only TT within target concentrations, respectively. Among 1010 (65.8%) transgender women who had neither hormone within target concentrations, 989 (64.5) had suboptimal E2 concentrations and 21 (1.4%) had supraphysiological E2 concentrations (geometric mean 655 pg/mL [range 213–3000 pg/mL]).

Transgender women who had any hormones within target concentrations were more likely to be younger, have lower body weight, have previously undergone orchiectomy, and were less likely to have multiple sex partners and HIV infection than those with neither hormone within target concentrations. The choice of combined or single hormone regimen and current use of antiretroviral agents were comparable between both groups.

### Factors associated with having hormone(s) within target concentrations at baseline

In multivariable logistic regression models, body weight of <60 kg (adjusted odds ratio [aOR] 1.43; 95% confidence interval [CI] 1.05–1.94, *p*=0.02) and prior orchiectomy (aOR 10.32; 95% CI 2.21–48.26, *p*<0.001) were significantly associated with having either hormone within target concentrations at baseline (Table 2). Gender identity, HIV status, and current use of combined hormone regimen were not significantly associated with having either hormone within target concentrations.

**Table 2.** Multivariable Analysis of Associated Factors of Having Hormone(s) Within Target Concentrations at Baseline

	Multivariable		
	aOR	95% CI	*p*
Age ≥25 years	0.76	0.51–1.14	0.19
Body weight <60 kg	1.43	1.05–1.94	0.02^*^
Gender identity			
Transgender women	Ref.		
Male	0.49	0.10–2.47	0.39
Female	2.67	0.58–12.23	0.21
Education less than bachelor's degree	1.20	0.84–1.72	0.31
Occupation			
Unemployed	Ref.		
Student	1.29	0.68–2.46	0.44
Employed (nonsex worker)	0.99	0.53–1.88	0.98
Sex worker	0.65	0.28–1.54	0.33
Underwent orchiectomy	10.32	2.21–48.26	<0.001^*^
HIV positive	1.09	0.78–1.51	0.63
Use combined hormone regimen	0.58	0.24–1.39	0.22

^*^ Indicates *p*-value of <0.05.

aOR, adjusted odds ratio; CI, confidence interval.

### Metabolic parameters between participants who had any hormones and those who had neither hormone within target concentrations

Among those with available baseline metabolic parameters (Table 3), median hematocrit (hematocrit: 40.3 [38.2–41.7] vs. 43.4 [41.1–45.5] %, *p*<0.001), hemoglobin (13.6 [12.8–14.3] vs. 14.6 [13.8–15.2] g/dL, *p*<0.001), and creatinine concentrations (0.78 [0.71–0.85] vs. 0.84 [0.77–0.92] mg/dL, *p*<0.001) were significantly lowered among transgender women who had any hormones within target concentrations compared with transgender women who had neither hormone within target concentrations.

**Table 3.** Baseline Metabolic Parameters of Transgender Women Included in the Analysis

Metabolic parameters	Overall	Hormone(s) within target concentrations	Neither hormone within target concentrations	*p*
	*n*=246	*n*=104	*n*=142	
Hematocrit, %	41.6 (39.6–44.3)	40.3 (38.2–41.7)	43.4 (41–45.5)	<0.001^*^
Hemoglobin, g/dL	14.2 (13.3–14.9)	13.6 (12.8–14.3)	14.6 (13.8–15.2)	<0.001^*^
Mean corpuscular volume, fL	82.8 (77.7–86.5)	83 (77.1–85.5)	82.3 (78.2–87.1)	0.61
White blood cells,×10^3^/μL	7.1 (5.8–8.2)	7.2 (6–8.6)	6.8 (5.6–8.1)	0.11
Platelet counts,×10^3^/μL	278.5 (239–320)	287.5 (238.5–328)	273 (240–317)	0.33
	*n*=542	*n*=206	*n*=336	
Creatinine, mg/dL	0.82 (0.74–0.89)	0.78 (0.71–0.85)	0.84 (0.77–0.92)	<0.001^*^
	*n*=454	*n*=183	*n*=271	
ALT, IU/L	16 (12–22)	15 (11–22)	17 (12–23)	0.09
	*n*=80	*n*=35	*n*=45	
Total cholesterol, mg/dL	201 (180.5–223)	201 (186.5–217)	201.5 (177.5–227)	0.82
HDL, mg/dL	60 (53–69)	65 (54–71)	58 (53–69)	0.21
LDL, mg/dL	133 (113–148)	128 (114–147)	136 (113–148)	0.18
Triglyceride, mg/dL	84 (66–121.5)	92 (71–123)	83 (62–120)	0.46

Data are shown in median (IQR).

^*^ Indicates *p*-value of <0.05.

ALT, alanine aminotransferase; HDL, high-density lipoprotein; LDL, low-density lipoprotein.

### Hormone concentrations at follow-up visit

A total of 302 (19.7%) transgender women came to at least one follow-up visit, and had both E2 and TT concentrations tested. Transgender women who came to follow-up visits were significantly more likely to identify themselves as female (16.6% vs. 4.5%, *p*<0.001) or to be unemployed (14.4% vs. 8.9%, *p*=0.02); and less likely to have undergone orchiectomy (0.7% vs. 2.9%, *p*=0.02) or have HIV infection (1.9% vs. 5.8%, *p*=0.01) at baseline. There were no significant differences in the proportion of transgender women who had any hormones within target concentrations at baseline between those who came and did not come to follow-up visits.

During 12 months of follow-up visit, 165 (54.6%) transgender women were able to achieve or maintain either hormone within target concentrations: 21 (7.0%) had both hormones, 9 (3.0%) had only E2, and 135 (44.7%) had only TT within target concentrations. All 137 transgender women who achieved neither hormone within target concentrations during follow-up period had normal male testosterone concentrations, and 7 transgender women (5% of 137) had supraphysiological E2 concentrations.

**FIG. 1.**
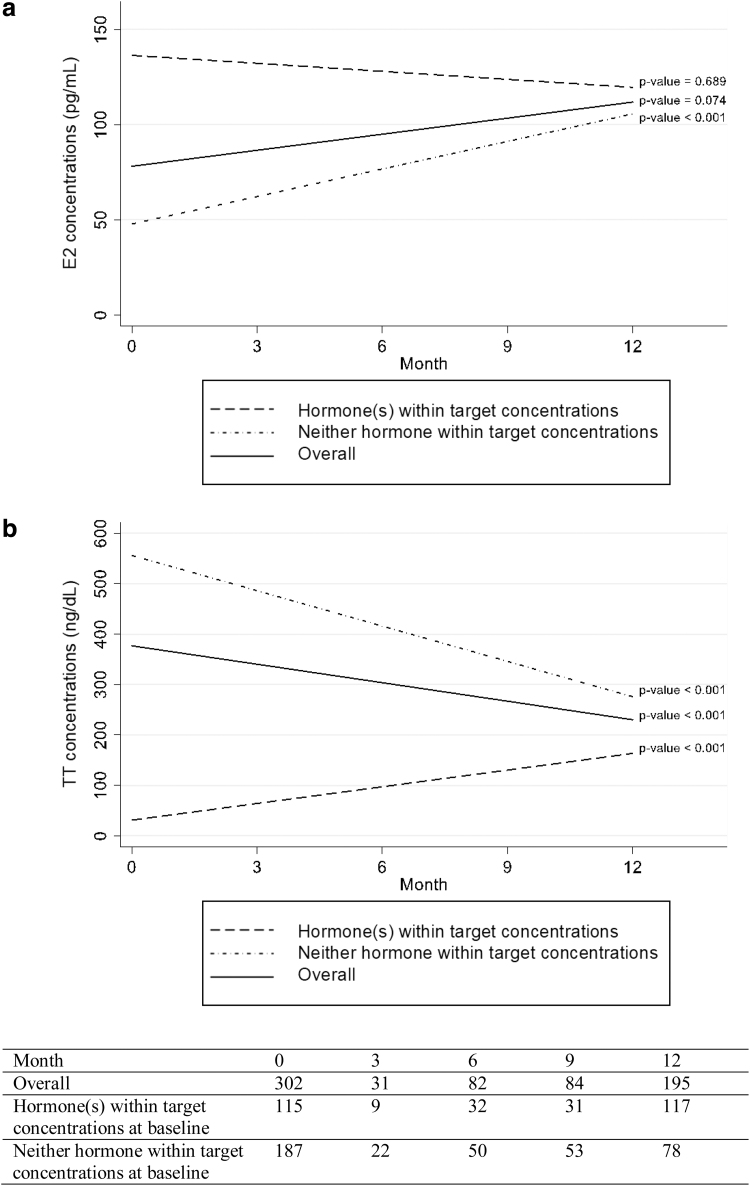
Linear trend changes of E2 **(a)** and TT **(b)** concentrations over follow-up visits at each follow-up visit categorized by those who had any hormones within target levels and had neither hormone within target concentrations at baseline. E2, estradiol; TT, total testosterone.

Among 187 transgender women who had neither hormone within target concentrations at baseline and came to at least one follow-up visit, 84 (44.9% of 187) were able to achieve any hormones within target concentrations during follow-up. Overall, there was an increasing trend of E2 concentrations and a decreasing trend of TT concentrations toward target concentrations during follow-up visits (Fig. 1).

## Discussion

In a real-world clinic setting, we demonstrated that one-third of Thai transgender women who had been using nonmedically supervised FHT had any hormones within target concentrations, and that E2 concentrations of the majority of transgender women were suboptimal rather than supraphysiological. Hematocrit, hemoglobin, and creatinine concentrations were the only metabolic parameters significantly different from those who had any hormones within target concentrations and those who did not. During a 12-month follow-up period, both E2 and TT showed a positive trend toward target concentrations, and approximately half were able to achieve or maintain at least one hormone concentration within target ranges, suggesting a positive effect from attending a comprehensive transgender-specific health care facility.

An estrogen-based regimen, either single or as part of a combined regimen, was the most common hormone regimen among Thai transgender women in our study (62.4%). From our experience, this is likely due to its low price. For example, the two most common regimens in our study (ethinyl estradiol + cyproterone acetate and estradiol valerate) cost <0.5 USD/pill in Thailand. In comparison, an antiandrogen such as cyproterone acetate costs 2 USD/pill, approximately four times higher than the estrogen-based regimen. Many Thai transgender women also take over-the-counter hormones based on their transgender peers' experience and recommendation, possibly leading to the continuing trend of using the estrogen-based regimen.

Because many Thai transgender women rarely seek medical advice on FHT use, this raises concerns about improper hormone concentrations, particularly supraphysiological concentrations. Our study highlighted that although two-thirds of transgender women did not achieve target concentrations before attending a health care facility, only 2% had supraphysiological E2 concentrations. On the contrary, more than half of transgender women endured suboptimal hormone concentrations at baseline. Although this is a less concerning matter from the safety perspective, it is plausible that many Thai transgender women would not reach the physical changes they are expecting and consequently unable to mitigate gender dysphoria.

Previous studies have reported the effect of FHT on metabolic laboratory values in transgender women. Decrements in hematocrit and hemoglobin concentrations among transgender women who were using FHT are well documented,^9–13^ and our findings are consistent with previous reports. These changes occur due to the influence of androgen on the erythrocytosis rate. The role of estrogen in erythropoiesis is less understood, but a strong impact is unlikely.^14^ Hematocrit can decrease to CGW physiological concentrations.^11^ These changes can occur as fast as 3 months after FHT initiation before reaching its stable levels.^9,13^ We demonstrated a 3% hematocrit difference between those who had hormones and those who had neither hormone within target concentrations, comparable with the previously reported 4% decrement. Although transgender women who were using FHT reported elevated platelet counts,^12,13^ the concentrations were still in the cisgender reference range.^14^ Our study found higher platelet counts among those who had any hormones than those who had neither hormone within target concentrations, though the difference was not significant.

In contrast to the impact of FHT on hematocrit and hemoglobin concentration, the effect on creatinine concentration is less established. Studies showed decreased creatinine concentrations among transgender women who were using FHT.^11–13^ However, the changes were minimal, and the concentrations were between the CGM and CGW reference range.^11^ Comparably, we found significantly lowered creatinine concentrations among those who had any hormones within target concentrations compared with those who had neither. Because total body muscle mass impacts creatinine concentrations,^15^ a decrement is expected among transgender women who were using FHT, especially those who achieved physical responses. However, we were unable to confirm this due to missing data. The clinical implication is still an issue, particularly for calculating glomerular filtration rate, which requires sex and creatinine concentration in the formulation. Therefore, the results must be interpreted with caution.

A study consisting of 16 transgender women in a U.S. community clinic showed that all transgender women managed to achieve target E2 concentrations, and 67% managed to achieve target TT concentrations 6 months after FHT initiation.^16^ In contrast, approximately half of our participants managed to achieve or maintain either hormone within target concentrations. Differences between clinical trials and real-world settings and the number of participants may have contributed to such results. Regardless, our findings demonstrated a positive trend toward target concentrations of both E2 and TT at follow-up visits, suggesting a positive effect from attending a comprehensive transgender-specific health care facility.

By using clinical data from a service-based real-world clinic setting rather than through a clinical trial, we encountered many missing data such as physical feminizing effects from FHT, dosages of the FHT, metabolic parameters, as well as losses to follow-up visits. These led us to an inability to conclude the longitudinal metabolic effect and clinical efficacy of FHT. Many transgender women who underwent orchiectomy were not included in the analysis because testosterone was not measured. Finally, other hormonal concentrations (e.g., free testosterone, sex hormone-binding globulin, luteinizing hormone, and follicle-stimulating hormone) were not measured. However, to our knowledge, this is among the largest studies to report information on hormone concentrations and related issues among transgender women who were using FHT in a real-world clinic setting.

## Conclusion

Our real-world setting study demonstrated that one-third of Thai transgender women who were using FHT had any hormones within target concentrations at baseline. Most transgender women who had neither hormone within target concentrations had suboptimal rather than supraphysiological E2 concentrations. At follow-up visits, there was a positive trend of both E2 and TT concentrations toward target concentrations. More than half managed to achieve or maintain at least one hormone concentration within target concentrations, suggesting a positive effect from attending a comprehensive transgender-specific health care facility.

## Notes

**Cite this article as:** Hiransuthikul A, Janamnuaysook R, Getwongsa P, Peelay J, Samitpol K, Amatsombat T, Chumnanwet P, Chancham A, Kongkapan J, Rueannak J, Himma L, Srimanus P, Teeratakulpisarn N, Avery M, Wansom T, Mills S, Ramautarsing RA, Phanuphak N (2022) Estradiol and testosterone concentrations among thai transgender women in a transgender-led, integrated gender-affirming care and sexual health clinic: a real-world analysis, *Transgender Health* 7:6, 539–547, DOI: 10.1089/trgh.2021.0049.

## Abbreviations Used

ALT: alanine aminotransferase
aOR: adjusted odds ratio
ART: antiretroviral therapy
ARVs: antiretroviral agents
CGM: cisgender men
CGW: cisgender women
CI: confidence interval
CPA: cyproterone acetate
DRSP: drospirenone
DTG: dolutegravir
E2: estradiol
EC: estradiol conjugate
EE: ethinyl estradiol
EH: estradiol hemihydrate
EV: estradiol valerate
FHT: feminizing hormone therapy
FTC: emtricitabine
GSD: gestodene
HDL: high-density lipoprotein
IQR: interquartile range
IRB: Institutional Review Board
LDL: low-density lipoprotein
LINKAGES: Linkages across the Continuum of HIV Services for Key Populations Affected by HIV
PEPFAR: President's Emergency Plan for AIDS Relief
PrEP: pre-exposure prophylaxis
TDF: tenofovir disoproxil fumarate.
TT: total testosterone
USAID: United States Agency for International Development
